# SPAC: a scalable and integrated enterprise platform for single-cell spatial analysis

**DOI:** 10.1186/s12859-025-06339-2

**Published:** 2026-01-29

**Authors:** Fang Liu, Rui He, Thomas Sheeley, David A. Scheiblin, Stephen J. Lockett, Lisa A. Ridnour, David A. Wink, Mark Jensen, Janelle Cortner, George Zaki

**Affiliations:** 1 Biomedical and Computational Science Directorate, Frederick National Laboratory for Cancer Research, Rockville, MD USA; 2 Essential Software Inc., Gaithersburg, MD USA; 3 Department of Biochemistry, Purdue University, West Lafayette, IN USA; 4 Optical Microscopy and Analysis Laboratory, Frederick National Laboratory for Cancer Research, Frederick, MD USA; 5 Cancer Innovation Laboratory, Center for Cancer Research, National Cancer Institute, National Institutes of Health, Frederick, MD USA; 6 Center for Biomedical Informatics and Information Technology, National Cancer Institute, Bethesda, MD USA

**Keywords:** Spatial omics, Spatial proteomics, Multiplex imaging, Single-cell analysis, High-performance computing, Interactive visualization, Tumor microenvironment, Scalable analysis

## Abstract

**Background:**

Advancements in spatially resolved single-cell technologies are transforming our understanding of tissue architecture and disease microenvironments. However, analyzing the resulting high-dimensional, gigabyte-scale datasets remains challenging due to fragmented workflows, intensive computational requirements, and a lack of accessible, user-friendly tools for non-technical researchers.

**Results:**

We introduce SPAC (analysis of SPAtial single-Cell datasets), a scalable, web-based platform for efficient and reproducible single-cell spatial analysis. SPAC employs a four-tier architecture that includes a modular Python-based analysis engine, seamless integration with high-performance computing (HPC) and GPU acceleration, an interactive browser interface for no-code workflow configuration, and a real-time visualization layer powered by Shiny for Python dashboards. This design empowers distinct user roles: data scientists can extend and customize analysis modules, while bench scientists can execute complete workflows and interactively explore results without coding. Built-in reproducibility features and collaborative workflow support ensure that analyses are transparent and easily shared across research teams. Using a 2.6-million-cell multiplex imaging dataset from a 4T1 breast tumor model as a benchmark, SPAC reduced unsupervised clustering time from ~3 hours on a CPU to under 10 minutes with GPU acceleration, achieving more than a 20-fold speedup. It also enabled fine-grained spatial profiling of distinct tumor microenvironment compartments, demonstrating the platform’s scalability and performance.

**Conclusions:**

SPAC addresses major barriers in single-cell spatial analysis by uniting an intuitive, user-friendly interface with scalable, high-performance computation in a robust and reproducible framework. By streamlining complex analyses and bridging the gap between experimental and computational researchers, SPAC fosters collaborative workflows and accelerates the transformation of large-scale spatial datasets into actionable biological insights.

**Supplementary Information:**

The online version contains supplementary material available at 10.1186/s12859-025-06339-2.

## Background

Spatial omics technologies, spanning spatial transcriptomics and proteomics, are providing unprecedented insights into the molecular and biological processes within tissue at single-cell resolution [1–5]. Specifically, for image-based protein profiling, multiplex imaging techniques such as imaging mass cytometry (IMC) [6], multiplexed ion beam imaging (MIBI) [7], multiplexed immunofluorescence (MxIF) [8], cyclic immunofluorescence (CyCIF) [9], and co-detection by indexing (CODEX) [10], allow simultaneous measurement of dozens to thousands of proteins in individual tissue sections. Computational pipelines, including HALO [11] and MCMICRO [12], leverage segmentation and pixel-level deep learning to delineate cell boundaries and extract per-cell features such as spatial coordinates, staining intensities, and morphological attributes. While these rich spatial datasets present new avenues for understanding tissue organization at high-resolution, they also introduce considerable analytical and computational challenges.

A critical barrier in leveraging these high-dimensional datasets is translating the detailed spatial data into actionable biological insights. Key analytical tasks include identifying distinct cellular subsets, characterizing cell–cell interactions, and uncovering spatial patterns linked to experimental conditions or clinical outcomes. Existing computational tools for spatial proteomics, such as Seurat [13], SPIAT [14], Giotto [15], Squidpy [16], and SCIMAP [17], primarily cater to bioinformaticians and researchers with extensive computational expertise. Consequently, laboratory scientists and clinicians lacking specialized backgrounds in image analysis, data science, statistics, or programming, face barriers when attempting independent data analysis and interpretation.

Additionally, the conventional division of roles within research teams further impedes efficient analysis workflows. Experimental biologists typically oversee hypothesis generation, experimental design, and data acquisition, whereas computational experts (e.g., bioinformaticians and data scientists) separately develop and maintain custom scripts and pipelines. This division frequently results in slow and fragmented workflows, where data are exchanged across disparate systems, analyses are performed on specialized computational setups, and outputs are often limited to static visualizations. Such compartmentalization can hinder iterative exploration, slow hypothesis testing, and compromise reproducibility. Without close involvement from the experimental team, general-purpose bioinformatics pipelines may be run without aligning analyses to the study’s hypotheses and decision points. SPAC addresses this by enabling bench scientists to configure and execute analyses themselves via question‑oriented templates and stratifications. 

High-resolution whole-slide imaging (WSI) compounds these computational challenges, producing datasets that may reach hundreds of gigabytes in size, comprising millions of cells over large tissue areas (e.g., a 50-plex, 4 cm^2^ slide at 0.3 μm resolution). Under these conditions, compute-intensive tasks become bottlenecks on standard workstations. For instance, graph-based clustering algorithms such as PhenoGraph [18] construct k-nearest neighbor graphs based on Euclidean distances using normalized cellular features, refine these graphs by adjusting edge weights according to the number of shared neighbors between cells, and then apply community detection algorithms (e.g., Leiden or Louvain clustering) to delineate cellular clusters. When combined with iterative parameter tuning and dimensionality reduction methods like UMAP [19], these analyses can require tens of gigabytes of memory for datasets containing more than 10 million cells and tens of markers. Consequently, there is an urgent need for platforms that integrate user friendly interfaces with scalable, high-performance computing or cloud-based resources.

**Fig. 1:**
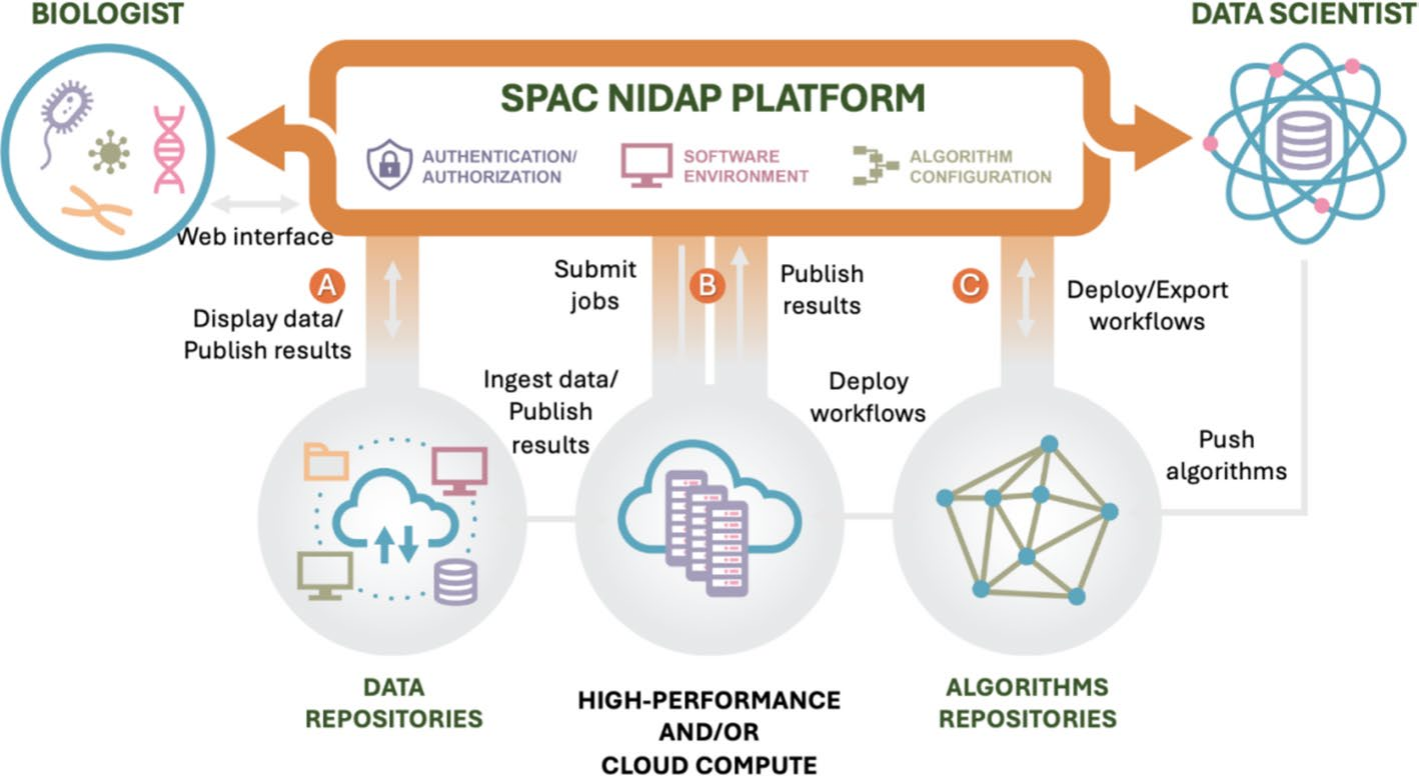
Overview of the SPAC infrastructure. SPAC is deployed on the NIH Integrated Data Analysis Platform (NIDAP) to support both bench biologists and data scientists. **A** Data ingestion and results publication. Biologists upload imaging-derived per-cell tables (e.g., CSV from HALO, MCMICRO, QuPath, Visiopharm) to NIDAP; SPAC converts them into AnnData and publishes derived outputs back to the platform. **B** Scalable computation. Compute-intensive steps (e.g., clustering, dimensionality reduction, spatial statistics) are dispatched to CPU/GPU nodes through the NIDAP HPC Connector; progress and logs are monitored and results are returned automatically, no direct HPC logins required. **C** Methods lifecycle. Data scientists push algorithms and workflows to SPAC repositories; validated workflows are deployed/exported to the interactive environment for reuse and sharing. Together, these connections enable web-based exploration of large-scale, HPC-powered analyses, from data sampling and preprocessing to clustering and spatial analysis, with transparent monitoring and shareable reports

To address these limitations, we developed SPAC (analysis of SPAtial single-Cell datasets), a spatial single-cell analysis ecosystem built on a four-tier architecture that combines community-standard and in-housing developed Python packages, scalable computation, and interactive exploration. Figure 1 provides an infrastructure map showing data ingestion (A), HPC offload/return (B), and workflow deployment (C). For the linear *user* workflow, see the text-based Supplementary Fig. 1. The four-tier architecture democratizes the analyses along four complementary dimensions: (i) Browser-based access: SPAC allows researchers to execute comprehensive analyses directly within a web browser environment, eliminating local software installations. This accessibility empowers bench biologist to easily launch workflows, tune parameters, and inspect results interactively without command-line expertise. (ii) Integrated pipeline: SPAC provides a modular, unified analytical framework that encompasses exploratory data analysis (EDA), quality control, clustering, phenotyping, and spatial analysis. This cohesive pipeline maintains a consistent data lineage, minimizes burdens associated with data format conversion, and ensures reproducibility by preserving a detailed, shareable record of all computational steps and parameters even as personnel change. (iii) Scalable HPC/cloud back-end: SPAC seamlessly connects to on-premises or cloud-based HPC resources, providing compute nodes with more than 150 GB of memory and specialized accelerators (e.g., GPUs). Automated load balancing, parallel computing, and containerized execution allow users of all experience levels to process large-scale datasets containing millions of cells rapidly and efficiently. (iv) Collaboration and reproducibility: SPAC’s architecture fosters a collaborative research environment where biologists can pose hypotheses and interact with dynamic visualizations (such as spatial maps, heatmaps, and boxplots), while data scientists refine and configure robust analytical pipelines. Integrated features such as version control, parameter tracking, and shared project workspaces facilitate transparent collaboration and robust reproducibility.

## Implementation

### SPAC ecosystem

**Fig. 2:**
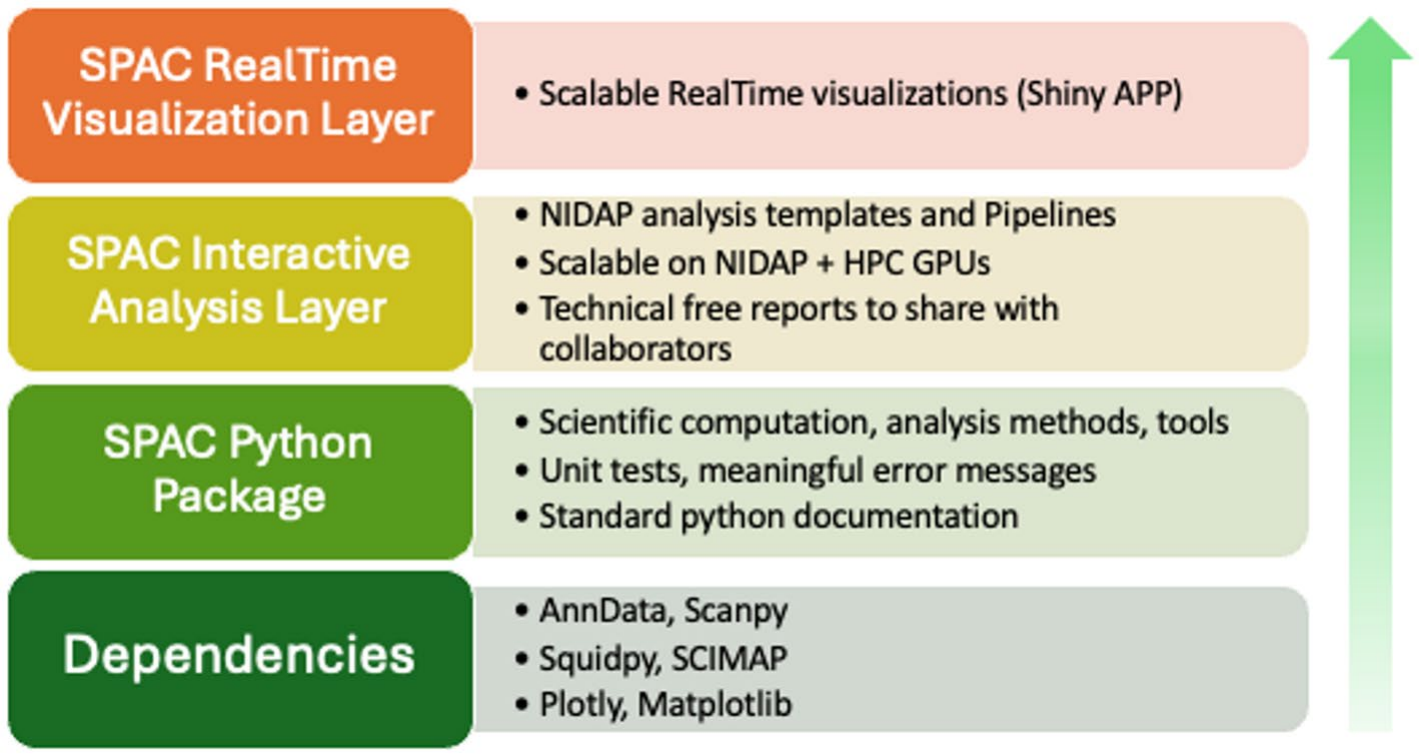
The SPAC ecosystem: A modular framework for scalable image analysis. SPAC has a four-layer architecture. The Foundational Dependencies layer integrates open-source tools for core single-cell and spatial proteomics methods. The SPAC Python package layer adds custom analysis routines, rigorous software practices, and GPU-enabled HPC support. The Interactive Analysis layer delivers web-based pipelines via NIDAP Code Workbook templates, scaling automatically to HPC resources and generating shareable reports. The Real-Time Visualization layer decouples heavy computations from the front end, enabling dynamic exploration of large spatial datasets through dashboards and live annotations

The SPAC ecosystem is designed with a modular, layered architecture that leverages community-standard tools while providing enterprise-level scalability, interactive pipelines, and real-time visualization (Fig. 2). Each layer in the stack is tailored to accommodate different user groups from software engineers and data scientists developing new workflows to bench scientists and principal investigators who primarily review and interpret results without installing or maintaining complex software.

#### Foundational dependencies

At the base of the SPAC stack lie established open-source packages for single-cell and spatial proteomics analysis, including AnnData [20], SCANPY [21], Squidpy, and SCIMAP. These tools, integral to the “scverse” [22] ecosystem, provide data structures and methods that have become de facto standards in Python-based single-cell analytics. The scverse ecosystem is a collaborative community-driven initiative that unifies diverse single-cell analysis tools into a coherent framework, promoting interoperability, reproducibility, and standardized workflows. By building on these foundational libraries, SPAC aligns with community best practices, ensures ongoing compatibility with widely adopted formats, and inherits robust core functionalities such as dimensionality reduction, clustering, and spatial graph construction.

#### SPAC python package

Directly built on top of these community tools, the SPAC Python package extends [23] and augments existing functionalities to meet the specific needs of multiplexed tissue imaging workflows. It implements specialized analysis routines that customize preprocessing of input datasets (e.g., batch correction, normalization, and data transformation), perform sample stratification, phenotyping, support HPC-enabled clustering schemes, and compute spatial statistics that are not available in standard packages. SPAC also supports the export of processed data, enabling researchers to perform independent downstream analyses using external tools. In addition, the package adheres to rigorous research software engineering principles by incorporating comprehensive unit tests, robust handling of edge cases, informative error messages, and standard Python documentation, all of which contribute to enhanced maintainability and reproducibility.

#### SPAC interactive analysis layer

The SPAC ecosystem can be deployed on any enterprise platform, such as Code Ocean [24], Galaxy [25], or Code Workbook from Palantir Foundry [26]. These platforms provide robust authentication and a configurable computational graph to manage workflows efficiently. For our deployment, we selected Code Workbook because of its modular building blocks that execute code, generate visualizations, output datasets, and record logs. For intramural NIH research scientists and their collaborators, the NIH Integrated Data Analysis Platform (NIDAP) [27], built on Palantir Foundry has been tailored for non-proprietary code and workflow development. By leveraging NIDAP’s Code Workbook templates and pipelines, the SPAC Interactive Analysis layer exposes the underlying SPAC Python package and its dependencies in a user-friendly, web-based environment. Within this framework, analysts can construct stepwise pipelines to ingest data, apply preprocessing or quality control, perform clustering or spatial analyses, and generate publishable figures. Key features include: (i) Web-hosted pipelines: configure parameters, run workflows, and monitor progress entirely through a browser interface, eliminating the need for local installations. (ii) HPC integration and scalability enhancements: SPAC seamlessly scales analyses to HPC environments with automated workload distribution for large datasets. In our implementation, we deployed SPAC on Biowulf [28], the on-premises NIH HPC cluster, which supports distributed or parallel execution (e.g., on GPU-enabled nodes). This ensures that computationally intensive steps, such as iterative clustering or large-scale image data processing, can be performed efficiently. (iii) Collaboration and reporting: compile final results into shareable, nontechnical reports that detail each analysis step and associated figures, promoting transparency and reproducibility across research teams. Outside NIDAP, SPAC’s pipelines are being packaged for Galaxy and published in the Galaxy Main ToolShed as the suite “suite_spac_tools”, allowing straightforward installation on any Galaxy instance (links provided in Availability and requirements).

#### SPAC real-time visualization layer

At the top of the stack, the SPAC Real-Time Visualization layer leverages Shiny for Python [29] and Posit Connect [30] to deliver on-the-fly interactive dashboards and exploratory figures. By decoupling heavy computations from the visualization front end, this layer empowers principal investigators, bench scientists, and other non-specialist stakeholders to quickly explore large spatial datasets, toggling between features or annotations and drilling down into specific regions of interest, while simultaneously generating live dashboards that create dynamic views, such as UMAP plots, hierarchical heatmaps, and neighborhood graphs, which update in real time as new data are loaded or parameters are adjusted. A hosted instance of the SPAC Interactive Visualization dashboard (built with Shiny for Python) is available on AppShare/Posit Connect for immediate, no-install exploration, and the dashboard’s source code is openly available (see the availability and requirements section).

This four-tier architecture establishes a clear separation of responsibilities between development and sharing. Software engineers and data scientists can concentrate on code quality and methodological innovation, while biologists and clinicians benefit from an intuitive, browser-based environment for data exploration and interpretation. In practice, image analysts or bioinformaticians design custom analyses using the SPAC Python package; core facility scientists or bench scientists run complex pipelines via the Interactive Analysis layer; and principal investigators rely on the Real-Time Visualization layer to review outcomes. By integrating open-source solutions with enterprise-ready HPC scaling, SPAC effectively streamlines the workflow from raw data to actionable insights in single-cell spatial proteomics research.

### Modules and NIDAP template

**Fig. 3:**
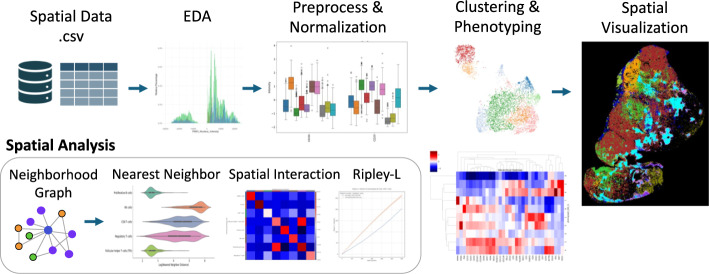
An overview of the SPAC workflow for single‐cell spatial analysis. The top row depicts the main pipeline steps: In the Spatial Data step, CSV files are imported, merged, and optionally downsampled or annotated. Exploratory Data Analysis (EDA) uses methods such as histograms and boxplots to reveal marker distributions and potential outliers. During Preprocessing & Normalization, techniques such as quantile scaling, arcsinh transformation, and batch correction are applied to standardize the data. Clustering & Phenotyping relies on algorithms such as PhenoGraph, t‐SNE, or UMAP for grouping cells by marker expression and assigning phenotypes. In Spatial Visualization, these phenotypes are color‐coded and overlaid on tissue images. The bottom row focuses on Spatial Analysis modules, including building a Neighborhood Graph to depict relationships among cells, performing Nearest Neighbor analyses to quantify infiltration or proximity, detecting Spatial Interactions between cell types, and calculating Ripley’s L to measure clustering or dispersion across distance scales. A Hierarchical Heatmap (on the right) integrates marker expression and phenotype relationships, providing deeper insights into subpopulations and their spatial patterns

An overview of the SPAC workflow for single‐cell spatial analysis is shown in Fig. 3. The default SPAC user workflow -depicted in Fig. 3, summarized in Supplementary Fig. 1, and accompanied by an executable NIDAP Code Workbook in Supplementary Fig. 2- encompasses data aggregation, multi-slide sampling, exploratory data analysis, feature preprocessing and normalization, clustering, dimensionality reduction, cluster analysis, and spatial analysis. A consolidated summary of supported inputs modalities and module functions is provided in Supplementary Table S1. In particular , quality control and normalization are configurable in the Preprocessing & Normalization module. The interface provides per-feature distributions and missingness and outlier checks, supports cell- and feature-level filtering, and includes normalization choices such as arcsinh transformation and quantile scaling, with optional batch-effect mitigation. SPAC retains both raw and normalized layers in the AnnData object to preserve data lineage.

We elaborate on four key modules—Key Data Structure & Formats; Visualization of Features & Annotations; Cell Phenotypes; States & Functions; and Spatial Analysis—that collectively streamline these steps into an efficient and reproducible analysis pipeline.

#### Key data structure and formats

In a multiplexed tissue imaging workflow, the initial data are generated by image-processing software (e.g., HALO, MCMICRO, Visiopharm [31], QuPath [32]) that performs cell segmentation and computes per-cell features such as spatial coordinates, protein staining intensities, and morphological descriptions (e.g., size, eccentricity). These measurements are exported as H5AD files or tabular data in CSV format, where each row corresponds to a single cell, and each column captures intensities, metadata, and positional attributes.

Users can batch-upload these files to the NIDAP platform, which aggregates multiple files into a single dataset while enforcing granular access control at both the group and the individual levels. Upon ingestion, SPAC converts these single-cell tables (and any optional annotations) into AnnData objects, a widely adopted data structure for single-cell genomics and proteomics. This standardized format facilitates the storage of multiple data versions by allowing raw, normalized, and annotated layers to coexist within a single object. It also enables seamless interoperability through integration directly with the broader single-cell Python ecosystem (e.g., SCANPY, Squidpy), thereby streamlining downstream analysis and visualization without cumbersome file transformations. Additionally, by retaining X–Y positional metadata, the format readily supports spatial operations such as neighborhood graph construction, distance computations, and interactive map overlays, ultimately ensuring consistency throughout preprocessing, clustering, and spatial analysis steps while simplifying collaboration and transitioning between exploratory and production workflows.

#### Visualization of features and annotations

In SPAC, two fundamental terms are used to describe the data: Features and Annotations. Features are measurable attributes of each cell, such as biomarker intensity. Annotations are categories assigned to cells to group them based on specific criteria. For example, annotations can denote cell type (e.g., “animal type” with labels such as “animal1” and “animal2”), spatial regions (e.g., “hypoxic” or “normal stroma”), or clusters derived from computational methods (e.g., “PhenoGraph”), thereby facilitating both biological interpretation and spatial analysis.

SPAC provides a comprehensive suite of integrated visualization tools that span the entire workflow from exploratory data analysis to final results presentation. Implemented using libraries such as Matplotlib, Plotly, and Seaborn, SPAC provides basic plots (e.g., histograms, boxplots, heatmaps, and scatter plots) to examine features and annotations, revealing underlying patterns, distributions, and relationships. Building on these standard charts, SPAC enables visualizations tailored to high-dimensional data, spatial statistics, and spatial organization. For example, dimensionality reduction techniques project complex, multi-feature data into lower-dimensional representations, thereby making it easier to discern overall similarities and differences among cells or samples. Hierarchical heatmaps with dendrograms, on the other hand, visualize the hierarchical clustering of features with annotation dimensions based on a computed correlation matrix, thereby elucidating the hierarchical structure of cellular subpopulations. These visualization modules are embedded directly into the analysis pipeline, ensuring that results at each stage can be immediately inspected and interactively explored without the need to export data to external software.

#### Cell phenotypes, states, and functions

A central objective in single-cell spatial proteomics is to identify and characterize diverse cell phenotypes. Multiplexed imaging platforms capable of measuring 10–60 markers per cell are well suited for dissecting heterogeneous cell populations from broad lineages (e.g., immune versus epithelial) to subtle functional states (e.g., signaling pathway activation or T‐cell exhaustion). SPAC supports two complementary strategies for phenotype determination: knowledge-based (manual gating) and data-driven (unsupervised clustering) approaches.

The knowledge-based phenotyping approach leverages well-established biomarkers (e.g., immunological or histopathological markers) associated with specific cell types and manually defines phenotypes by setting intensity-based thresholds. Marker expressions are categorized into binary states (e.g., ‘0’ or ‘1’) to represent phenotypic classes such as “CD4-negative” versus “CD4-positive,” allowing precise, context-specific annotation of cell populations. SPAC can also consolidate multiple biomarker expressions into a single composite phenotype_code. For example, a regulatory T-cell phenotype can be succinctly defined as CD4 +CD25 +FOXP3 +, integrating simultaneous expressions of multiple markers into one standardized code. This flexibility enables domain experts to annotate specific cell subtypes or functional states without custom programming, simply by specifying a human-readable phenotype_name and the corresponding composite phenotype_code. By providing standardized and reproducible phenotype definitions, this feature enhances scalability and ensures consistency across extensive, multi-slide datasets, which is particularly valuable in high-throughput experimental contexts.

Complementing this manual approach, SPAC supports unsupervised clustering algorithms, such as PhenoGraph, to systematically uncover novel cell populations based purely on phenotypic similarity. PhenoGraph employs a three-step process: it first normalizes the marker intensity data to ensure comparability across cells, then constructs a k-nearest-neighbor (KNN) graph by identifying the k nearest neighbors for each cell using Euclidean distance, and subsequently refines the graph by adjusting the edge weights so that the weight between any two cells scales with the number of neighbors they share. Finally, graph clustering–using either the Louvain or Leiden algorithm–is applied to delineate distinct cell communities. With adjustable clustering resolutions, this method can reveal subtle subpopulations often missed by manual gating. Additionally, dimensionality reduction techniques (e.g., t-SNE or UMAP) provide visual summaries of high-dimensional data, facilitating inspection of emergent clusters and guiding hypothesis generation. These unsupervised methods can identify phenotypic clusters associated with biological or clinical outcomes such as patient prognosis, thus enabling refined patient stratification beyond conventional clinical classifications.

By integrating manual gating methods anchored in established biomarker knowledge with flexible unsupervised clustering techniques, SPAC provides a robust and versatile framework that examines known cell phenotypes across various states or conditions and simultaneously discovers novel subpopulations, advancing our understanding of tissue organization and disease mechanisms.

#### Spatial analysis

SPAC streamlines the analysis of spatial organization within multiplexed tissue images across multiple scales, from nearest neighbor distances to larger scale clustering patterns. It integrates spatial analytic methods with intuitive visualization tools and systematically interrogates spatial relationships among cell phenotypes, such as assessing phenotype arrangements, identifying co-location or exclusion patterns, and detecting significant cell–cell interactions beyond random chance.

Nearest Neighbor analysis quantifies the proximity of a specified “source” phenotype relative to other cell types, highlighting patterns of local adjacency or avoidance. To assess broader spatial patterns, particularly when comparing case versus control conditions, SPAC implements Ripley’s L statistic [33], which measures whether two phenotypes exhibit spatial aggregation, dispersion, or random distribution across varying spatial scales, where the random distribution is modeled using a Poisson point process under the assumption of homogeneous tissue distribution. SPAC builds Neighborhood graphs using Squidpy by connecting cells via *k*‐nearest neighbors or radius‐based criteria with customized “edge correction” [23], computes a Cluster Interaction Matrix which tallyies the edges between distinct phenotypes, and applies permutation‐based Neighborhood Enrichment scores to determine whether phenotype pairs co‐locate significantly more or less frequently than expected by chance. Neighborhood enrichment and interaction matrices can be stratified by user-defined annotations (e.g., region, timepoint) and are visualized with faceted plots and downloadable result tables for downstream use.

In addition, SPAC includes a customizable neighborhood profiling framework that captures the local cellular environment around each cell. This feature summarizes the composition of a cell’s immediate neighborhood, providing a high-resolution context for each cell. Such neighborhood profiles enable flexible and scalable downstream analyses, for example, by generating a spatial UMAP embedding [34] that groups cells by the similarity of their microenvironments or by performing infiltration profiling to quantify how certain cell types penetrate different tissue regions. By characterizing each cell’s neighborhood across different samples or conditions, researchers can interpret differences in spatial organizatio with unprecedented resolution, gaining deeper insight into tissue structure and cell–cell interactions. Spatial plotting capabilities, both static and interactive, enable visualization and refinement of spatial distributions within defined tissue contexts. The interactive spatial plot, built with Plotly Express, supports real‐time exploration features, such as zooming, on-hover annotations, and pinned color mapping for consistent and customizable phenotype labeling, facilitating clear interpretation and shareable visualizations. Together, these analytic and visualization tools form a comprehensive suite for dissecting complex spatial topographies within multiplexed tissue samples.

### HPC connector for scaling

**Fig. 4:**
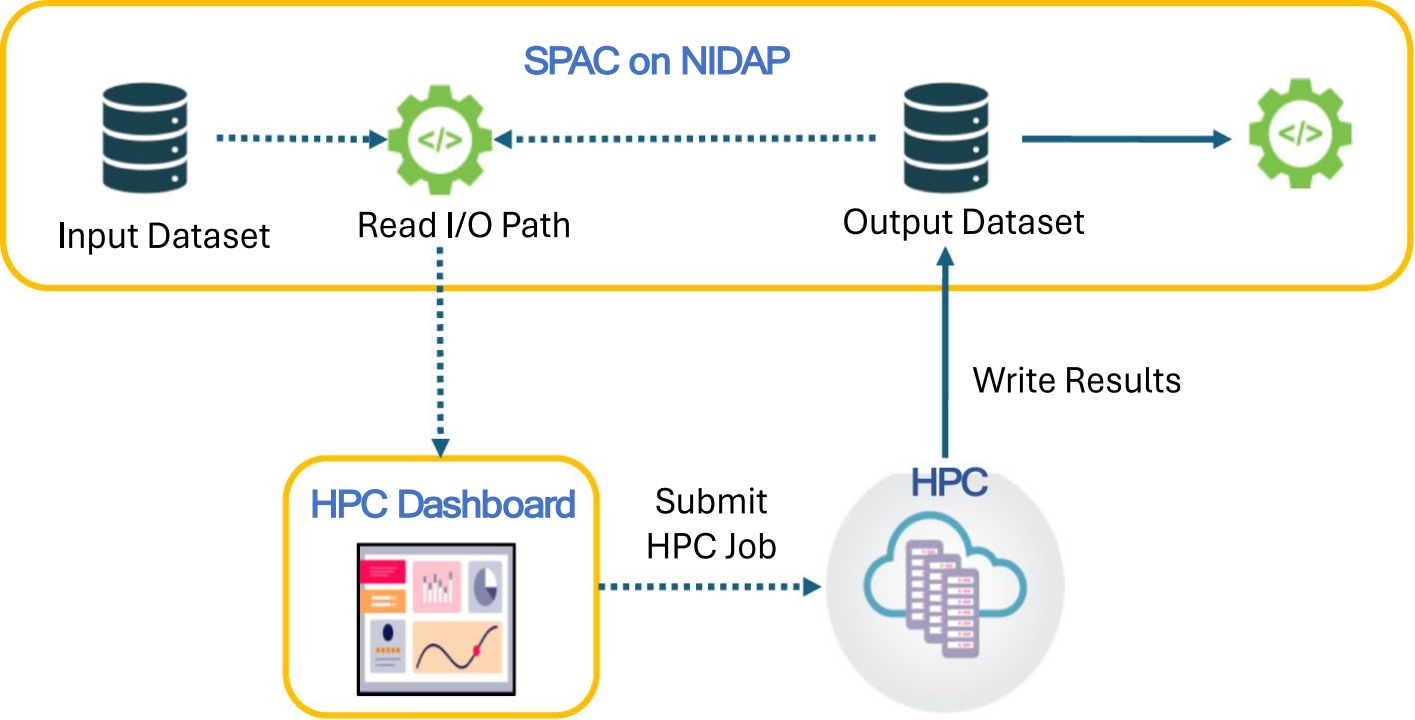
Scaling single‐cell analysis on HPC clusters via the NIDAP HPC Connector. SPAC leverages the NIDAP HPC Connector to offload large single‐cell analyses to HPC clusters. A secure service account reads input data from the SPAC pipeline on NIDAP, and then submits the job to GPU or high‐memory CPU nodes via the HPC Dashboard. The HPC Dashboard provides real‐time monitoring of job status, resource utilization, and logs. Once computations are complete, results are automatically returned to NIDAP for further visualization and downstream analysis. This workflow enables large‐scale computational tasks without requiring users to have direct HPC credentials or command‐line interaction

A core objective of SPAC is to address large‐scale computational demands by seamlessly offloading resource‐intensive analyses to HPC clusters. The NIDAP HPC Connector integrates HPC resources directly into the interactive analysis layer, allowing any user-configured computational steps–such as PhenoGraph clustering or other advanced analyses–to be executed on an HPC node (CPU or GPU). When a computationally demanding task is initiated, the connector automatically identifies the corresponding input data within NIDAP, dispatches the job to the appropriate HPC resource, and monitors job status in real time via an integrated HPC Dashboard. Upon completion, results are seamlessly returned to the interactive interface, enabling rapid, large‐scale analyses without requiring specialized HPC credentials or command‐line interaction. Example deployments use Slurm, a widely used open-source workload manager (e.g., NIH Biowulf). The NIDAP HPC Connector is scheduler-agnostic and forwards analysis parameters to the local scheduler; exact job specifications and authentication mechanisms are site-specific. Figure 4 illustrates the workflow, and Supplementary Fig. 3 maps the UI parameters (partition, CPUs, memory, time, optional GPUs).

#### Infrastructure for multithreading and parallel processing

Internally, the HPC Connector leverages substantial computational resources available on institutional clusters (e.g., the Slurm-managed NIH Biowulf cluster) to distribute large workloads across multiple nodes or GPUs. For example, a PhenoGraph clustering job that might take an hour (or potentially fail) on a desktop environment can be completed in under ten minutes on GPU nodes. This approach not only scales to datasets comprising millions of cells but also provides a robust foundation for future integration of advanced machine learning and algorithmic tasks.

#### Usability

From the user’s perspective, HPC scaling is simplified to selecting a desired compute mode (CPU or GPU) within the Code Workbook interface. The HPC Connector processes the request and submits it to the appropriate HPC partition (e.g., “quick,” “norm,” or “GPU”) with the necessary resources (e.g., core count, memory, time). Logging, error checking, and intermediate file handling are automatically relayed back to the user’s Code Workbook node, ensuring a streamlined experience without requiring direct HPC account management.

#### Speed

**Fig. 5:**
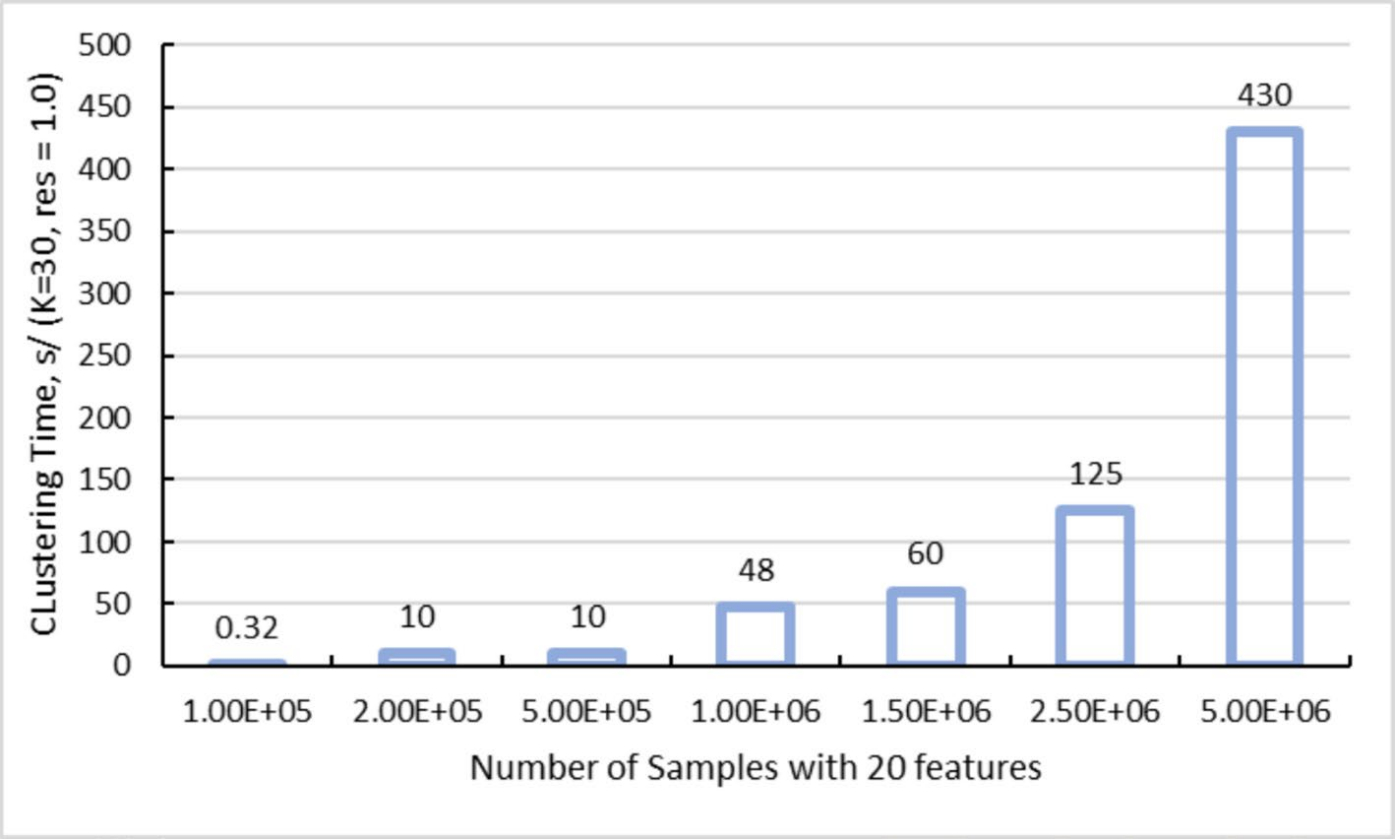
GPU‐accelerated PhenoGraph clustering times on synthetic datasets (k = 30, resolution = 1.0). The bar chart illustrates how clustering runtime scales when applying GPU‐accelerated PhenoGraph to synthetic single‐cell datasets ranging from 100,000 to five million cells, each with 20 features. The parameter k = 30 specifies the number of nearest neighbors used to construct the graph, while resolution = 1.0 determines the granularity of cluster detection. Even at the upper limit of five million cells, total processing time remains on the order of minutes, demonstrating near‐linear scalability and underscoring the efficiency gains from leveraging HPC resources

By leveraging powerful cluster nodes with up to terabytes of memory [28] and extensive GPU arrays, the HPC Connector significantly reduces execution times for large SPAC jobs. Bottlenecks typically associated with single-threaded operations, such as clustering, dimensionality reduction, and spatial interaction statistics, are mitigated through effective parallelization. Benchmarks on synthetic datasets (up to five million cells with approximately 20 features each) demonstrate near-linear scalability when running PhenoGraph with k = 30 (the number of nearest neighbors) and resolution = 1.0 (cluster granularity), as shown in Fig. 5. This setup enables users to explore multiple clustering parameters within a single session while maintaining rapid execution times.

#### Security

The HPC Connector employs a “service account” framework to ensure data integrity and security. Instead of requiring personal HPC credentials, the NIDAP platform securely manages job submissions and file transfers via established, audited APIs. All data transfers occur within a controlled environment using encrypted connections, thereby preventing unauthorized access to sensitive research data. This approach upholds enterprise‐level security while providing the scalable compute infrastructure essential for spatial single‐cell analysis.

### Animals and tumor model

Female BALB/c mice (8–10 weeks old; RRID:MGI:2683685) were obtained from the Frederick Cancer Research and Development Center and housed five per cage under standard conditions. Each mouse received a subcutaneous injection of 200,000 4T1 triple-negative breast-cancer cells (ATCC; RRID:CVCL_0125) into the fourth mammary fat pad. Tumor measurements began one week after tumor cell injection, using a Vernier caliper and calculated in cubic millimeter volumes according to the following equation:

$$\left[ {\left( {\text{short diameter}} \right)^{{2}} \times {\text{long diameter}}} \right]/{2}$$

To delineate hypoxic regions, pimonidazole (PIMO) was administered intravenously (60 mg/kg) 30 min before euthanasia. Mice were euthanized 15 min in CO2 per the requirements of the ACUC protocol and committee, and tumor tissues were harvested on days 9, 19, and 30 post-injection and flash-frozen for downstream multiplex imaging. The protocol used to label, acquire, and analyze images is provided in Additional file 4.

## Results

To evaluate SPAC’s performance on real-world data, we applied the platform to multiple collaborative research projects, using hundreds of slides and tens of millions of cells. Specifically, we analyzed a single-cell dataset (~ 2.6 million cells) derived from multiplex immunofluorescence (MxIF) images of BALB/c mice bearing 4T1 tumors collected on days 9, 19, and 30 post-injection. The images were processed in HALO to generate per-cell expression tables, which served as input for SPAC (see Section “Animals and tumor model”). Each cell was characterized by its spatial coordinates, intensities for eight biomarkers (Hif1a, NOS2, COX2, β-catenin, vimentin, E-cadherin, Ki67, aSMA) as well as PIMO nucleus and cytoplasm intensities, morphological features (e.g., cytoplasm area, nucleus roundness), and localization within three distinct tumor regions: tumor normoxia (median oxygen level: 6.8%), tumor hypoxia (median oxygen level: 1.3%) [35], and necrotic areas. Whole tissue slides were collected from eight animals at three time points (days 9, 19, 30) after tumor cell injection. Raw data are provided in Additional file 3. An end-to-end analysis was performed using the SPAC Interactive Analysis layer workbook along with the SPAC Interactive Dashboard for real-time visualization and sharing of results. This analysis demonstrates SPAC’s versatility in characterizing cell phenotypes and analyzing spatial distribution in multiplex imaging data, with additional spatial interaction, biological interpretation, and statistical analyses to be presented elsewhere.

### GPU Acceleration of Unsupervised Clustering and Optimization

Given the computational challenges outlined earlier, GPU resources provide advantages for tasks dominated by matrix operations and graph‐based optimizations (e.g. PhenoGraph clustering, Louvain clustering, and UMAP dimensionality reduction). In this study, an unsupervised PhenoGraph clustering task on 2.6 million cells with nine biomarkers was accelerated from approximately 2.5 hours on an AMD EPYC 7543-based CPU node to about 7 min on an NVIDIA A100 GPU node using the Grapheno implementation [36]. The CPU run utilized an average of 7.5 cores and 13 GB of memory. This resulted in a ~20-fold speedup, highlighting the efficiency of GPU acceleration for large-scale clustering tasks.

A key factor in the GPU’s performance is its streamlined workflow. The implementation begins with a single k-nearest neighbors (KNN) calculation that constructs a connectivity graph by efficiently capturing local similarities in high-dimensional cell data. This graph then serves as the basis for four separate Louvain clustering procedures with different resolution parameters. Because of inherent parallelism and efficient GPU memory management, running multiple clustering resolutions adds only minimal overhead compared with computing a single resolution independently. Thus, the entire process from the KNN calculation through all clustering steps is completed in a few minutes, highlighting the substantial acceleration achieved over traditional CPU-based methods.

**Fig. 6:**
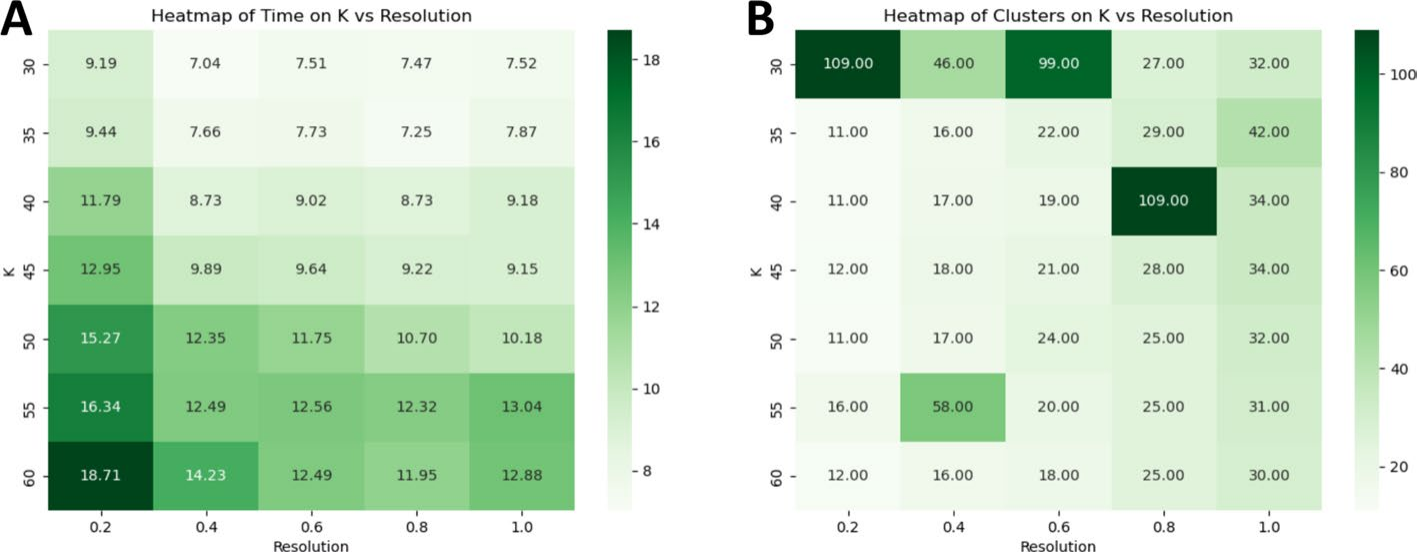
GPU‐accelerated PhenoGraph tuning across k-neighbors and resolution. (**A**) Heatmap of runtime (minutes) across combinations of PhenoGraph parameters k (vertical axis) and resolution (horizontal axis), using ~2.6 million cells with nine biomarkers. Darker green cells indicate longer runtimes, although all configurations remain under 20 min. (**B**) Corresponding heatmap of the total number of clusters produced. In this parameterization, a larger numeric resolution value yields a more granular partition, producing more clusters. These results highlight SPAC’s ability to leverage HPC GPU resources and systematically explore the parameter space with interactive turnaround times, enabling fine‐grained analysis of tumor‐associated cell populations

To optimize cluster granularity, we systematically varied the PhenoGraph parameters: the number of neighbors (k) and the resolution, which controls the coarseness of clustering. SPAC enables GPU-accelerated *k*/resolution parameter sweeps for PhenoGraph with interactive turnaround times (see Figs. 5 and 6), allowing users to tune cluster granularity directly from the browser. As illustrated in Fig. 6, SPAC’s HPC pipeline enables rapid exploration of these parameter settings. The runtime heatmap (Fig. 6A) shows that all combinations of k and resolution complete in under 30 min on a GPU, while the cluster‐count heatmap (Fig. 6B) reveals that higher resolution values generally yield more clusters. By leveraging GPU acceleration and parallel computing, SPAC allows users to test multiple configurations within a single session.

### Phenotype annotation

**Fig. 7:**
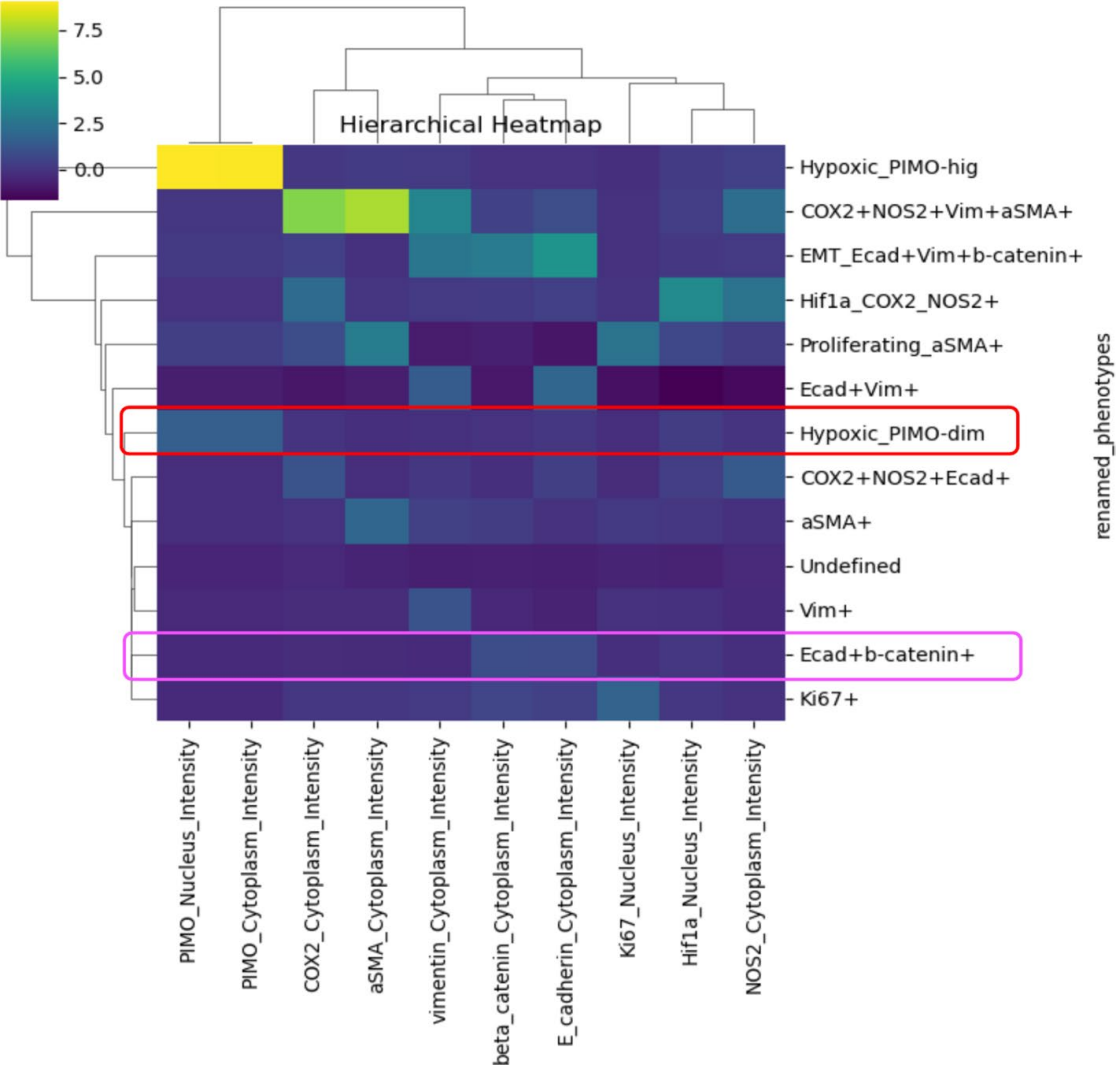
Hierarchical heatmap of renamed phenotypes (rows) clustered by marker intensities (columns). Each cell represents the scaled expression level (blue = lower expression; yellow = higher expression) of a particular marker within a given renamed phenotype. Dendrograms indicate similarity groupings for both markers and phenotypes, revealing distinct subpopulations based on their shared expression patterns. Clusters 4 and 15 (outlined in magenta in supplementary Fig. 4) are merged into the “Ecad + b-catenin + ” phenotype. Clusters 0 and 7 (outlined in red in Supplementary Fig. 4) are form the “Hypoxic_PIMO-dim” phenotype

The outputs from PhenoGraph clustering across various parameter settings were evaluated using a hierarchical heatmap of biomarker expression and a UMAP visualization, alongside expert domain knowledge. The optimal condition (k = 35, resolution = 0.6) yielded 16 clusters, a choice driven by the balance between granularity and interpretability. Specifically, this setting effectively captured distinct cellular subpopulations while minimizing noise and over-segmentation. Each of the 16 clusters exhibited a unique biomarker expression profile, which was consistent with established cellular phenotypes, thereby facilitating annotation and renaming for downstream analysis. For example, the hierarchical heatmap (Supplementary Fig. 4), which displays z-score normalized marker expressions, revealed that clusters 4 and 15 marked with magenta boxes because their uniquely high levels of E-cadherin and β-catenin. These clusters were consequently merged and annotated as “Ecad + b-catenin + ”. Similarly, clusters 0 and 7, marked by red boxes, exhibited moderate PIMO expression, a hypoxia marker, these clusters were combined and renamed “Hypoxic_PIMO-dim”. The final annotated phenotypes are presented in the hierarchical heatmap in Fig. 7, where the merged phenotypes are outlined again in the same color scheme to facilitate direct comparison.

### Spatial distribution of cellular phenotypes across tumor regions

To further illustrate SPAC’s capabilities, we leveraged the SPAC Interactive Dashboard to investigate the spatial distribution of cellular phenotypes across tumor regions. We hypothesized that epithelial cells characterized by high expression of E-cadherin and β-catenin are predominantly localized within tumor normoxic regions, reflecting the epithelial nature of 4T1-induced tumors. In contrast, cells annotated as “Hypoxic_PIMO-dim” were expected to reside primarily in tumor hypoxic regions.

**Fig. 8:**
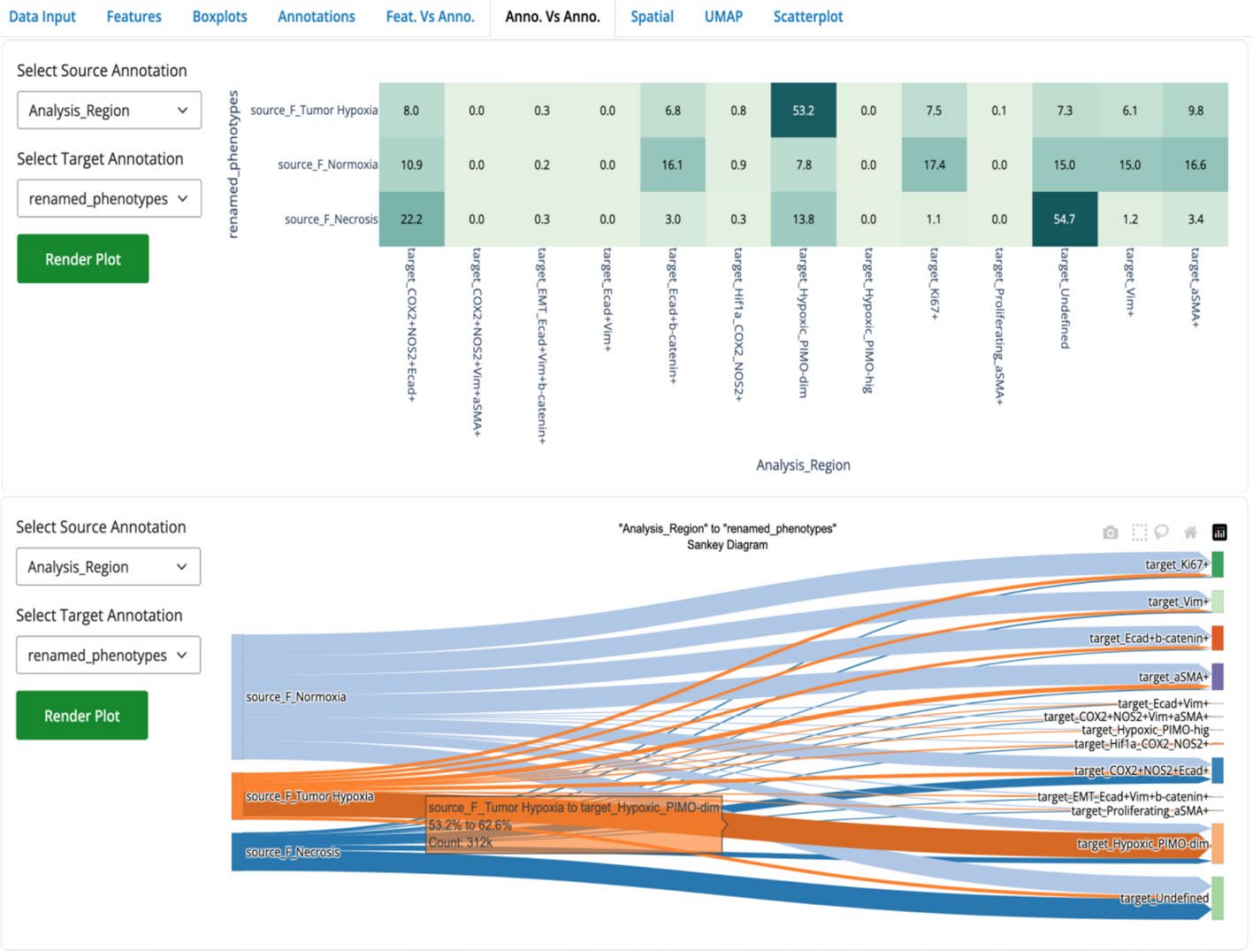
Relational heatmap and Sankey plot depicting the distribution of cells from different tumor regions (source annotation) across renamed cellular phenotypes (target annotation). In the Relational Heatmap (top), each cell of the matrix displays the percentage of cells (row total = 100%) that share both a specific tumor region (e.g., Hypoxia, Normoxia, Necrosis) and a given phenotype (e.g., Hypoxic_PIMO-dim, E-cadherin/β-catenin). Notably, 53.2% of cells in tumor hypoxic regions are Hypoxic_PIMO-dim, while E-cadherin/β-catenin–positive cells comprise approximately 16.1% of the normoxic compartment. The Sankey plot (bottom) visualizes these same relationships as proportional flows, indicating, for example, that 53.2–62.6% of all Hypoxic_PIMO-dim cells originate from hypoxic regions (~312,000 cells). Both the heatmap and Sankey diagram are generated by SPAC’s interactive dashboard, enabling dynamic exploration of cellular annotations and spatial context

Two complementary visualization tools were deployed. First, the Relational Heatmap employs color gradients to depict the frequency distribution between categorical annotations (i.e., tumor region and cellular phenotype). For instance, the heatmap (Fig. 8, top) indicates that 53.2% of cells in tumor hypoxic regions are classified as Hypoxic_PIMO-dim, whereas 16.1% of cells in tumor normoxic regions express E-cadherin/β-catenin. Second, the Sankey plot provides a flow diagram illustrating the proportional connectivity between tumor regions and phenotype annotations. In this display (Fig. 8, bottom), between 53.2 and 62.6% of Hypoxic_PIMO-dim cells originate from tumor hypoxic regions (representing approximately 312,000 cells), thereby reinforcing the expected spatial segregation. All analyses presented here were performed on a combined dataset comprising all animals.

**Fig. 9:**
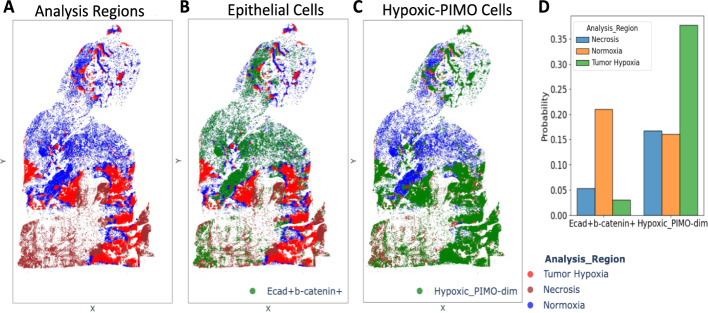
Spatial distribution of E-cadherin/β-catenin expressing epithelial cells and hypoxic_PIMO cells across different tumor regions on day 30. **A** Three tumor regions (normoxia, hypoxia, and necrosis) are delineated for spatial analysis. **B** E-cadherin + β-catenin + epithelial cells (green) predominantly localize in the normoxic region (blue), indicating higher oxygen levels. **C** Hypoxic_PIMO-dim cells (green) are enriched in the hypoxic (green) and necrotic (red) regions, reflecting reduced oxygen availability. **D** Bar chart summarizing the proportions of E-cadherin + β-catenin + and Hypoxic_PIMO-dim cells across the three tumor regions. The results highlight distinct spatial patterns for epithelial and hypoxic cell populations on day 30

SPAC’s ppatial plots enable the projection of cellular phenotypes onto the original spatial coordinates of tissue sections, offering both static and interactive modes for visualizing single-cell distributions. The interactive spatial plot provides dynamic user-driven exploration of cell populations across multiple annotations, facilitating deeper biological interpretation in real time. As illustrated in Fig. 9, three distinct tumor regions–normoxic, hypoxic, and necrotic–are defined in panel A. Panel B highlights epithelial cells co-expressing E-cadherin and β-catenin, which localize predominantly within normoxic areas, whereas panel C shows “Hypoxic_PIMO-dim” cells enriched in hypoxic and necrotic regions. In panel D, the bar chart quantifies these distributions on day 30, revealing that “Hypoxic_PIMO-dim” cells are predominant in hypoxic regions, whereas the epithelial population remains largely confined to normoxic regions.

This overlay analysis underscores SPAC’s ability to delineate tumor subregions based on distinct cellular phenotypes, an observation consistent with the underlying biology of the 4T1 model, where epithelial markers typically localize to normoxic areas, whereas hypoxic regions preferentially harbor PIMO associated hypoxia markers. Notably, SPAC supports the simultaneous display of multiple annotations (e.g., region labels and cell phenotypes) within each spatial plot, along with user-defined pin colors and stratification options. Such capabilities remain challenging to many existing tools, but are essential for capturing the complexity of tumor microenvironments and facilitating intuitive, high-throughput analysis.

Collectively, these findings demonstrate that SPAC robustly integrates phenotypic annotation with high-resolution spatial visualization, providing a powerful tool for deciphering the complex spatial organization of tumor tissues. This integrated approach not only streamlines analysis but also lays the groundwork for deeper insights into tumor biology and microenvironmental interactions.

## Discussion

SPAC was developed to meet the growing need for integrative, standardized computational tools to analyze, visualize, and interpret the spatial context at the cell-type level. By combining a modular software architecture, an interactive web‐based interface, and robust HPC integration, SPAC streamlines end‐to‐end analysis through intuitive graphical workflows that are accessible to both computational experts and experimentalists. Interactive dashboards provide real-time visual feedback, enabling immediate parameter tuning and facilitating data sharing among researchers.

In a case study analyzing a 4T1 breast cancer dataset of 2.6 million cells, SPAC demonstrated significant performance and scalability gains. GPU acceleration cut clustering runtimes from hours to minutes, and the platform’s real-time visualization enabled clear delineation of tumor subregions (e.g., distinguishing between normoxic and hypoxic regions based on their distinct E-cadherin/β-catenin expression patterns). This not only validated SPAC’s computational efficiency but also illustrated its ability to derive biological insights into tumor microenvironment heterogeneity.

Beyond this case study, SPAC has been deployed in collaborations with multiple research groups at the National Cancer Institute, processing more than 15 datasets from5 tissue and cancer types across diverse studies. These efforts underscore the platform’s versatility and its success in bridging the gap between data analysts and experimental biologists. Notably, the SPAC analysis library has been integrated into external applications (e.g., a Shiny-based interface), and contributions from the Purdue Data Mine (https://datamine.purdue.edu) have further extended its functionality. Collectively, this growing adoption highlights how SPAC fosters a more integrated and collaborative approach to spatial single-cell analysis in varied research settings.

Looking forward, we are actively developing several enhancements, including support for spatial transcriptomics (ST) data (e.g., Visium HD [37] and Slide-Seq [38], expanded statistical analyses for case–control comparisons, and additional clustering algorithms with further GPU acceleration. Because SPAC stores data in AnnData and builds on the scverse ecosystem, the same QC and normalization controls generalize to spatial transcriptomics, and we will expose ST-oriented defaults and filters in the provided templates. We also aim to introduce advanced spatial interaction metrics and refined batch-effect corrections to improve biological signal detection. An especially exciting direction is the integration of high-resolution tissue visualization via a framework like Vitessce [39], which will unify downstream analyses with whole-slide images in a web-accessible dashboard. These future extensions will further streamline data interpretation and broaden SPAC’s applicability.

## Conclusion

SPAC delivers an efficient, scalable, and user‐friendly solution for spatial single‐cell proteomics and is readily extensible to other spatial omics modalities. By coupling GPU-accelerated HPC with real-time, browser-based visualization, the platform supports rapid parameter exploration and detailed spatial characterization of cellular phenotypes. These capabilities are particularly valuable in clinical settings that compare primary tumors, metastases, or genomically distinct samples and seek to relate spatial context to immune infiltration and therapy response. By bridging experimental and computational expertise within a single reproducible workspace, SPAC accelerates biological discovery and sets the stage for spatial single-cell research.

## Availability and requirements

**Project name:** SPAC.

**Project homepage:** https://github.com/FNLCR-DMAP/SCSAWorkflow

**Real-time dashboard (Shiny for Python)—source code:** https://github.com/FNLCR-DMAP/SPAC_Shiny

**Hosted Shiny for Python app (AppShare/Posit Connect):** https://appshare.cancer.gov/spac-interactive-visualization/

**Galaxy tool wrappers:** Available on the Galaxy Main ToolShed as the suite **“suite_spac_tools”**(https://toolshed.g2.bx.psu.edu/view/fnlcr-dmap/suite_spac_tools) and are installable on any Galaxy instance.

**A step-by-step Jupyter tutorial** demonstrates installation, uploading, and a complete example run, with code and pinned environments to enable local reproduction. (https://github.com/FNLCR-DMAP/SCSAWorkflow/tree/main/paper/examples).

**Operating system(s):** Platform-independent

**Programming language:** Python

**Other requirements:** Python 3.9.13, NumPy 1.26.4, pandas 1.5, AnnData 0.10, Scanpy 1.9, and Matplotlib 3.9.2; a complete, version-pinned environment.yml is provided in the repository.

**License:** MIT License.

**Any restrictions to use by non-academics:** None.

## Supplementary Information

Supplementary Material 1

Supplementary Material 2

Supplementary Material 3

Supplementary Material 4

Supplementary Material 5

## Data Availability

The raw per-cell data supporting this study are provided in Additional file 3. The complete SPAC source code, processed example datasets, and reproducible notebooks are openly available at https://github.com/FNLCR-DMAP/SCSAWorkflow/. Additional files or clarifications can be obtained from the corresponding author upon reasonable request.
