# Supplementary material for: SPAC: a scalable and integrated enterprise platform for single-cell spatial analysis
Source: BMC Bioinformatics. 2026 Jan 29;27:25. doi: 10.1186/s12859-025-06339-2 (PMC12857135; doi:10.1186/s12859-025-06339-2)
Supplement: Supplementary file 1 — Supplementary Material 1 [file 12859_2025_6339_MOESM1_ESM.pptx]

## Slide 1
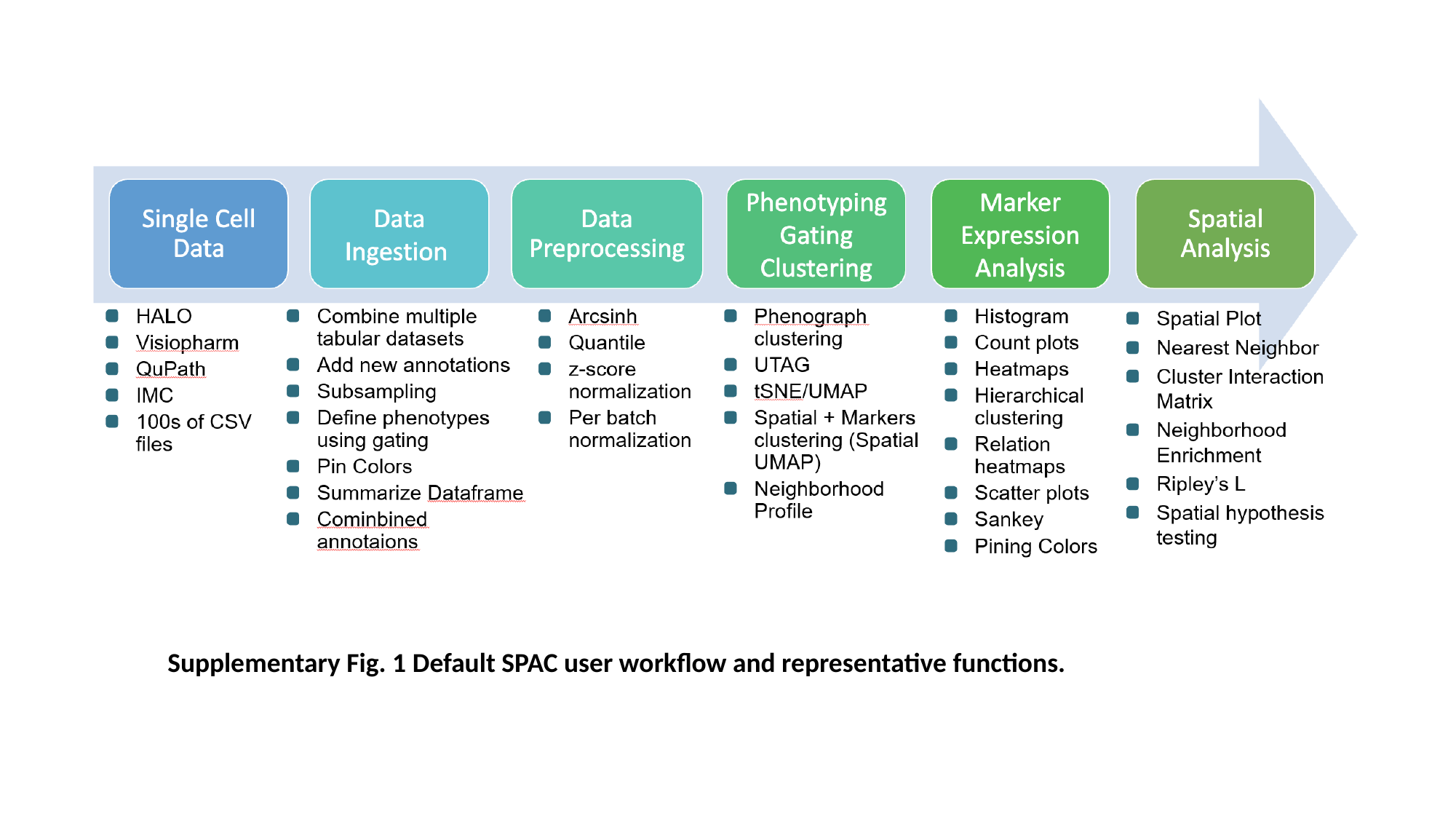

Supplementary Fig. 1 Default SPAC user workflow and representative functions.

## Slide 2
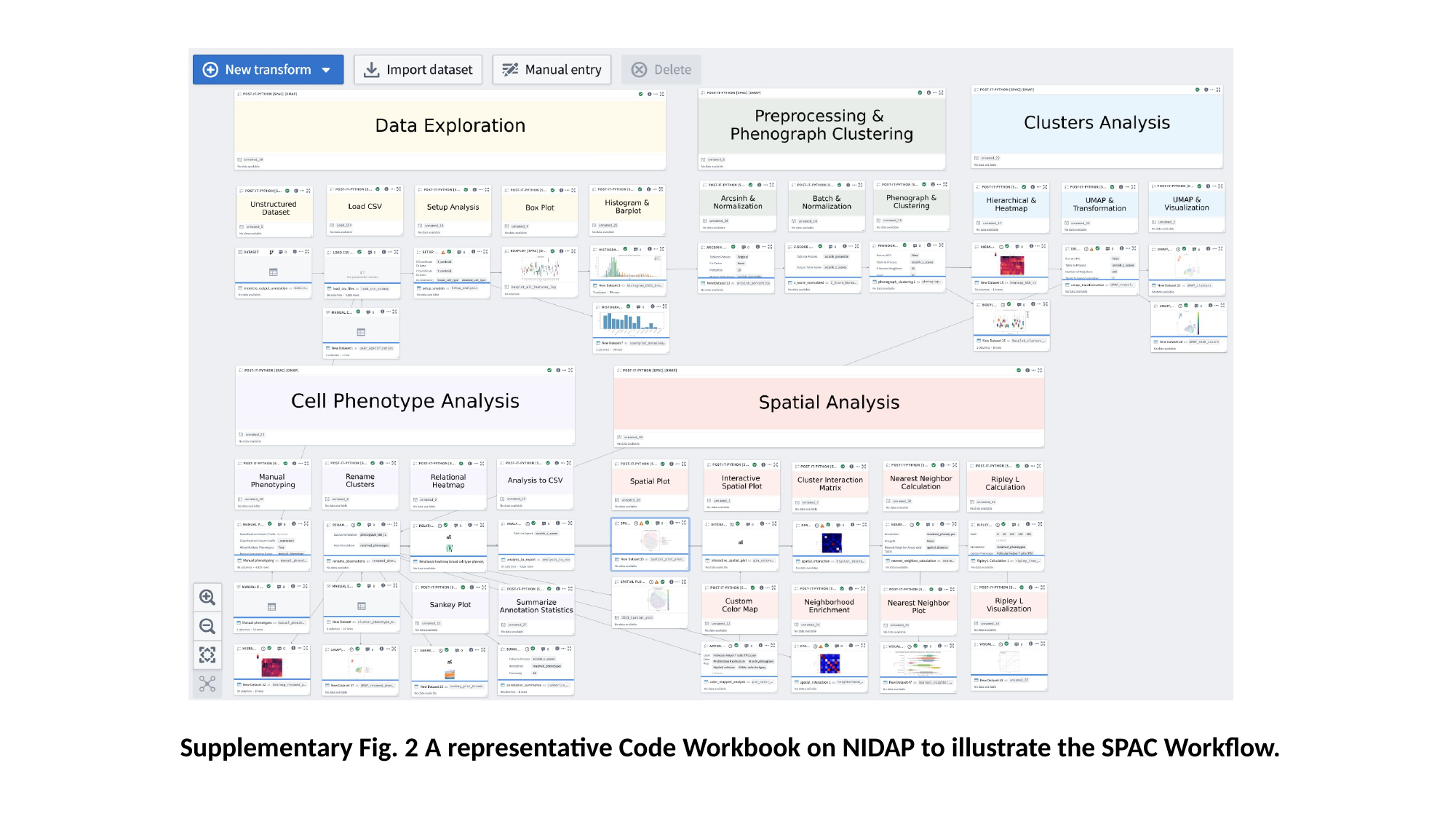

Supplementary Fig. 2 A representative Code Workbook on NIDAP to illustrate the SPAC Workflow.

## Slide 3
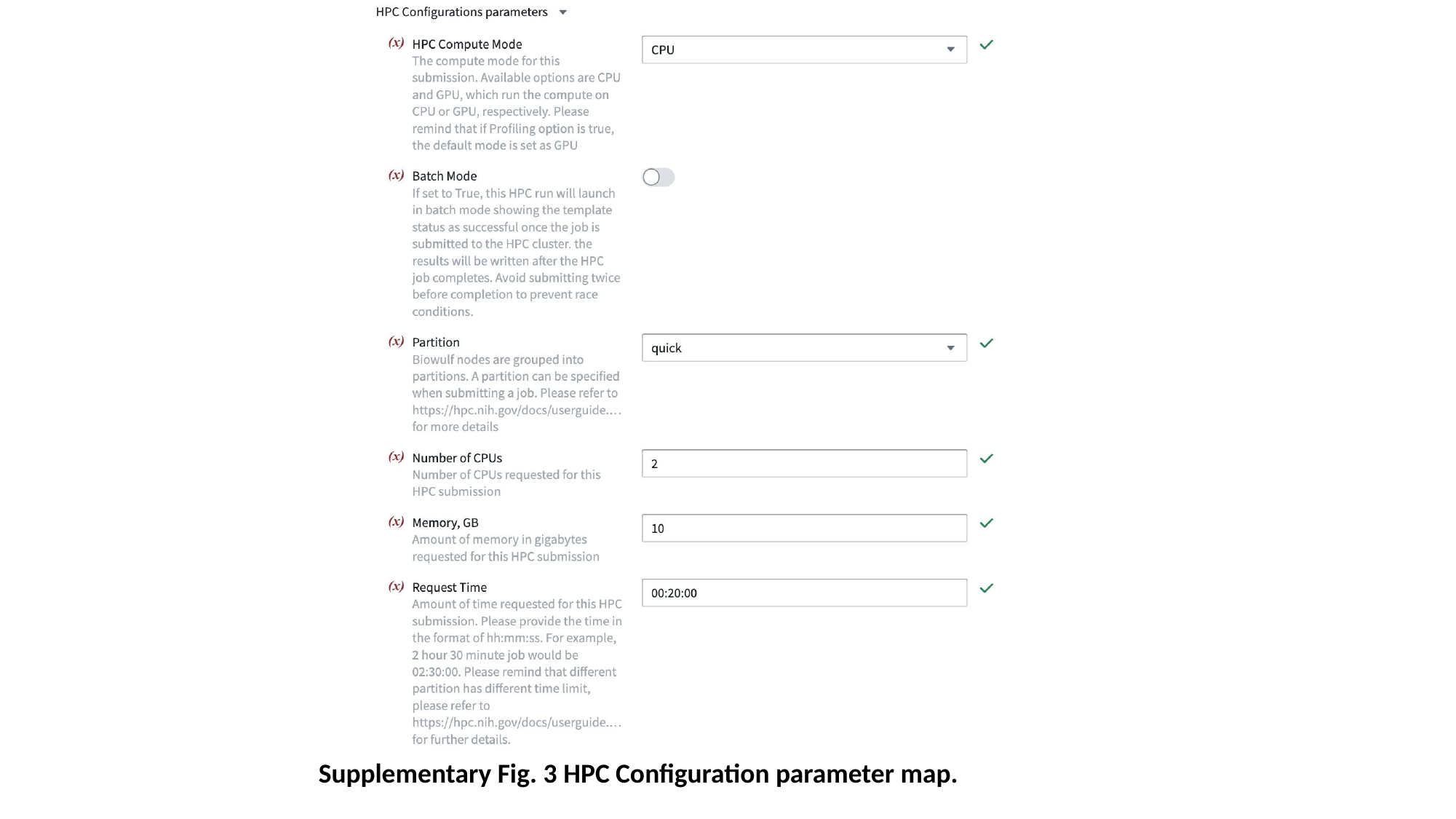

Supplementary Fig. 3 HPC Configuration parameter map.

## Slide 4
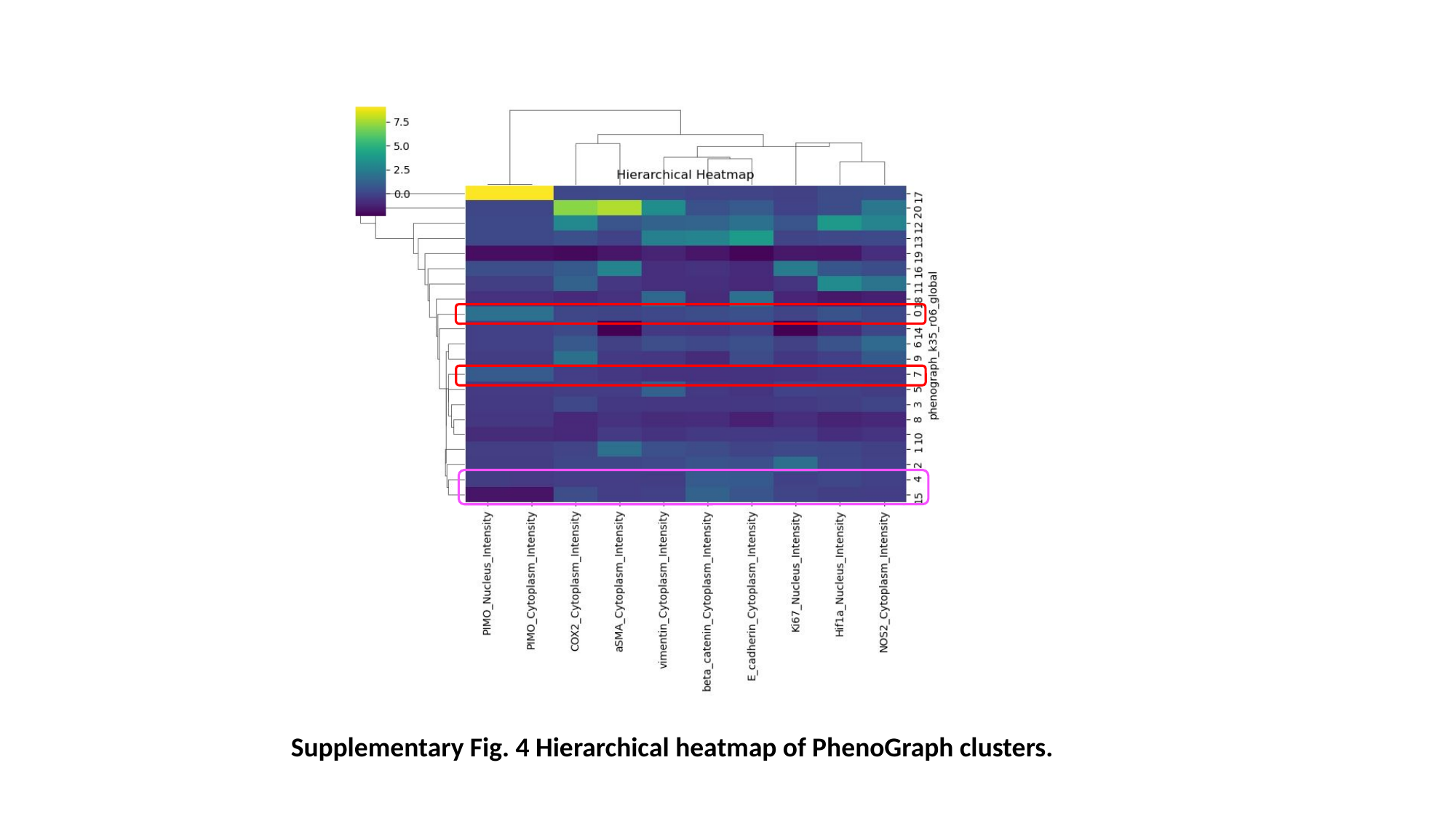

Supplementary Fig. 4 Hierarchical heatmap of PhenoGraph clusters.
