# Supplementary material for: SPAC: a scalable and integrated enterprise platform for single-cell spatial analysis
Source: BMC Bioinformatics. 2026 Jan 29;27:25. doi: 10.1186/s12859-025-06339-2 (PMC12857135; doi:10.1186/s12859-025-06339-2)
Supplement: Supplementary file 2 — Supplementary Material 2 [file 12859_2025_6339_MOESM2_ESM.docx]

**Supplementary Information**

**Supplementary Fig. 1 Default SPAC user workflow and representative functions.** The ribbon summarizes the recommended sequence from Single‑Cell Data → Data Ingestion → Data Preprocessing → Phenotyping/Gating/Clustering → Marker Expression Analysis → Spatial Analysis. Bullets indicate representative operations at each step: importing CSV/H5AD from HALO, MCMICRO, Visiopharm, or QuPath; combining datasets and adding annotations; applying quantile scaling, arcsinh transformation, and optional batch normalization; performing PhenoGraph clustering with UMAP/t‑SNE visualization; generating heatmaps, histograms, count plots, and hierarchical clustering; and running spatial modules such as interactive spatial plots, cluster interaction matrices, and neighborhood enrichment. These steps are implemented in NIDAP Code Workbook templates and can offload compute‑intensive components to CPU/GPU resources via the HPC Connector. The figure complements Supplementary Fig. 2, which shows the same workflow as an executable template.

**Supplementary Fig.2** **A representative Code Workbook on NIDAP illustrates the SPAC workflow** from data aggregation and sampling, exploratory data analysis, feature normalization, clustering, dimensionality reduction, and phenotype annotation to spatial analysis in a unified, modular environment, ensuring consistent data lineage and minimizing format conversion burdens. Each analysis step produces downloadable .csv files and figures ready for scientific presentation. Integrated HPC Connector in PhenoGraph and UMAP modules enable seamless offloading of computations to GPU/CPU resources for efficient processing of large-scale datasets. An example analysis of normal lymph node tissue demonstrates NIDAP’s standardized yet flexible design, enabling bench scientists to configure parameters, launch workflows, and view results in real time without command line expertise, while data scientists can refine robust pipelines, with reproducibility and transparency maintained through version control, parameter tracking, and shared project workspaces.

**Supplementary Fig. 3 HPC configuration parameter map in SPAC’s Interactive Analysis Layer.** The control panel collects the minimal scheduler parameters required for reproducible job submission on Slurm‑class clusters. Compute mode (CPU/GPU) selects the target resource type; Batch mode toggles asynchronous execution (marking the step complete once the job has been submitted); Partition maps to the Slurm queue/partition; Number of CPUs sets cpus‑per‑task; Memory, GB sets mem; and Request time sets the wall‑time limit (time, HH:MM:SS). The example values shown (CPU, quick partition, 2 CPUs, 10 GB, 00:20:00) illustrate a small test run. The UI validates entries, renders a structured job specification, and forwards it to the submission layer described in section 2.3; results and logs are streamed back to the user interface (see Fig. 4)

**Supplementary Fig. 4 Hierarchical heatmap illustrating the expression profiles of key markers (columns) across PhenoGraph clusters (rows).** Each cell represents the z‐score of a given marker’s intensity (yellow= higher expression; purple=lower expression). The dendrograms show how clusters and markers group together based on similarity in expression patterns, revealing distinct subpopulations. Clusters 4 and 15, outlined in magenta boxes, exhibit high E-cadherin and β-catenin expression. Clusters 0 and 7, outlined in red boxes, display moderate PIMO expression. These highlighted clusters were subsequently merged and renamed in the final analysis (see Fig. 7).
