# Supplementary material for: SPAC: a scalable and integrated enterprise platform for single-cell spatial analysis
Source: BMC Bioinformatics. 2026 Jan 29;27:25. doi: 10.1186/s12859-025-06339-2 (PMC12857135; doi:10.1186/s12859-025-06339-2)
Supplement: Supplementary file 4 — Supplementary Material 4 [file 12859_2025_6339_MOESM4_ESM.docx]

**Multiplex Immunofluorescent Staining and Image Acquisition**

Flash frozen tumor samples were embedded in Optimal Cutting Temperature (Tissue-Tek®O.C.T. Compound, Electron Microscopy Sciences, 62550-01, Hatfield, PA) and sectioned on a cryostat to 10μm thick. Sectioned tissues on slides were fixed in 4% paraformaldehyde (Electron Microscopy Sciences, 15710, Hatfield, PA) for 30 mins and then washed in 1X PBS 3x for 5 mins. Slides were then placed in blocking buffer (3% BSA (Bovine Serum Albumin, Sigma Aldrich, A2153, St.Louis, Missouri) in 1X PBS with 0.3% Triton X-100 (Electron Microscopy Sciences, 9410, Hatfield, PA)) for 1 hour at room temperature. Slides were then labeled with primary antibody at desired dilution (please see antibody table) in a humidified chamber overnight at 4 degrees Celsius. The next day all steps were done in the dark slides were washed 3x 10 min in 1X PBS. Slides are then stained with DAPI for 30 mins at room temperature. Slides were then washed 3x 10 mins in 1X PBS at room temperature. Slides were sealed with 1X PBS and Wirosil (BEGO, 52001, Lincoln, RI) around that edge which allowed for coverslip removal for continues steps. Samples were then imaged using a Nikon Ti widefield microscope equipped with a lumencor LED light system (395, 470, 555, 640), Plan Apo 20x DIC 0.75NA objective, a filter wheel with the following filters (Red FF01-600/52, GFP FF01-514/44, DAPI FF01-432/36), a 89000 Sedat Quad dichroic (444-479nm, 499-541nm, 567-625nm, 649-∞nm) and an Andor Zyla VSC-04649 sCMOS camera. Camera exposures and LED light power were determined visually dependent on the biomarker expression to get significant signal to noise for further analysis. After imaging slides coverslips were removed by peeling off the Wirosil (BEGO, 52001, Lincoln, RI) and placing the slides in fresh 1X PBS. Fluorophores were then chemically inactivated using a solution of 1:3:1 of Sodium Bicarbonate (Sigma Aldrich, S6014, St.Louis, Missouri) 1M at pH 10, DI Water, 30% hydrogen peroxide (Sigma Aldrich, H1009, St.Louis, Missouri) for 30 minutes. Slides were then washed in 1X PBS 3x for 10 min to remove residual bubbles and chemical inactivation solution. Slides then were resealed and imaged. DAPI will not be chemically inactivated, but all fluorophores should be quenched. After confirmation of chemically inactivating current fluorophores, coverslips were removed, and another set of antibody staining was conducted. All steps were repeated from fixation on as described above until all the biomarkers of interest have been labeled. A tissue section from each slide was labeled for 9 biomarkers (iNOS, Cox2, HIF1α, Ki67, α-smooth muscle actin, CD31, e-cadherin, vimentin and β-catenin) plus DAPI using the multiplex immunofluorescent staining protocol above. An adjacent section was labeled for α-smooth muscle actin, CD31 and reduced pimonidazole plus DAPI, because the pimonidazole staining did not chemically photobleach.

Antibody table:

| **Antibody Name** | **Company** | **Clone** | **Conjugate** | **Catalog Number** | **Dilution** |
| --- | --- | --- | --- | --- | --- |
| iNOS | Abcam | EPR16635 | AF555, AF568, AF647 | ab209595,  ab209027 | 1:100 |
| Cox2 | Cell Signaling Technologies | D5H5 | AF488 | 13596S | 1:100 |
| HIF1α | Abcam | EPR16897 | AF488, AF647 | ab208419, ab208420 | 1:100 |
| Ki67 | Cell Signaling Technologies | D3B5 | AF488, AF647 | 11882S, 12075S | 1:50 |
| αSmooth Muscle Actin | eBioscience(ThermoFisher) | 1A4 | eFluor 570 | 41-9760-82 | 1:400 |
| CD31 | Biolegend | MEC13.3 | AF488, AF594, AF647 | 102514, 102520, 102516 | 1:100 |
| E-cadherin | Cell Signaling Technologies | 24E10 | AF488, AF647 | 3199S, 9835S | 1:100 |
| Vimentin | Cell Signaling Technologies | D21H3 | AF488, AF555, AF647 | 9854S, 9855S, 9856S | 1:50 |
| β-Catenin | Cell Signaling Technologies | L54E2 | AF647 | 4627S | 1:200 |
| Pimonidazole | Hypoxyprobe | 11.23.22R | FITC | HP6-100Kit | 1:100 |

**Image Analysis**

Images were converted to ome.tiff to be used in the HALO software (Indica Labs, Albuquerque, New Mexico). Each image set of 4 images were registered in HALO using the image registration tool. Nuclei were segmented using a custom nuclei segmentation algorithm in the HALO AI module using a training data set generated by us. Nuclear size was limited to between 5 and 400 mm squared. The cytoplasm was defined as a 1.5 mm wide ring around each nucleus and the cell size were limited to between 10 and 600 mm squared. The fluorescence signal from each marker was quantified in the cytoplasm (or nucleus in the case of Ki67 and HIF1α) of each cell using the HighPlex FL v4.1.3 module in Halo.  The data was exported via .csv files to be used in SPAC.
