# Supplementary material for: SPAC: a scalable and integrated enterprise platform for single-cell spatial analysis
Source: BMC Bioinformatics. 2026 Jan 29;27:25. doi: 10.1186/s12859-025-06339-2 (PMC12857135; doi:10.1186/s12859-025-06339-2)
Supplement: Supplementary file 5 — Supplementary Material 5 [file 12859_2025_6339_MOESM5_ESM.docx]

**Supplementary Table S1. SPAC inputs/modalities and modules/functions**

| Section | Entry | Details | Examples / Defaults |
| --- | --- | --- | --- |
| Inputs & modalities | **Source software & file types** | Per‑cell features (marker intensities), XY coordinates, metadata | Software: HALO, MCMICRO, QuPath, Visiopharm; Files: CSV, H5AD; Modalities: MxIF, CyCIF, CODEX, IMC, MIBI |
| Modules | **Ingest & EDA** | Merge/multi‑slide sample; histograms, boxplots, spatial scatter | Dataset‑level summaries to assess distributions and batch effects |
| Modules | **Preprocessing** | Quantile scaling; arcsinh transform; optional batch correction | Defaults surfaced in templates; selected per dataset characteristics |
| Modules | **Clustering & DR** | PhenoGraph; UTAG; UMAP/t‑SNE | k‑NN and resolution parameters exposed; GPU/HPC support |
| Modules | **Phenotyping** | Knowledge‑based rules; cluster rename/merge | Curated threshold lists; composite phenotype codes |
| Modules | **Spatial analysis** | Neighborhood graph; nearest‑neighbor proximity; interaction enrichment; Ripley’s L | Radii/k tunable; permutation‑based enrichment |
| Modules | **Reporting & export** | Relational heatmap; Sankey; spatial plots; CSV/H5AD export | Pinned colors; annotation cross‑walks for reuse |

**Abbreviations:** DR, dimensionality reduction; k‑NN, k‑nearest neighbors; HPC, high‑performance computing; IMC, imaging mass cytometry; MIBI, multiplexed ion beam imaging; MxIF, multiplex immunofluorescence.

**Supplementary Table S1.** SPAC inputs/modalities and modules/functions. Inputs/modalities lists common source software and file types with required per‑cell content and example imaging modalities. Modules/functions summarizes the end‑to‑end workflow modules, representative functions, and typical defaults exposed in the user interface.
